# Sudden Cardiac Death-Etiology, Risk Factors and Demographic Characteristics: An Extensive Study of 1618 Forensic Autopsies

**DOI:** 10.3390/diseases12080168

**Published:** 2024-07-25

**Authors:** Ioana Radu, Anca Otilia Farcas, Victoria Nyulas, Carmen Corina Radu, Klara Brinzaniuc

**Affiliations:** 1 Doctoral School of Medicine and Pharmacy, George Emil Palade University of Medicine, Pharmacy, Science and Technology of Targu Mures, 540142 Targu Mures, Romania; ioanaradu888@gmail.com; 2 Department of Forensic Medicine Emergency County Hospital, “Constantin Opriș” Baia Mare, 430031 Baia Mare, Romania; 3 Department of Cell Biology, George Emil Palade University of Medicine, Pharmacy, Science and Technology of Targu Mures, 540139 Targu Mures, Romania; 4 Department of Informatics and Medical Biostatistics, George Emil Palade University of Medicine, Pharmacy, Science and Technology of Targu Mures, 540139 Targu Mures, Romania; 5 Institute of Forensic Medicine, 540141 Targu Mures, Romania; carmen.radu@umfst.ro; 6 Department of Forensic Medicine, George Emil Palade University of Medicine, Pharmacy, Science and Technology of Targu Mures, 540139 Targu Mures, Romania; 7 Department of Anatomy, George Emil Palade University of Medicine, Pharmacy, Science and Technology of Targu Mures, 540139 Targu Mures, Romania; klara.brinzaniuc@umfst.ro

**Keywords:** sudden cardiac death, forensic autopsy, demographic characteristics, CAD

## Abstract

Background: Sudden cardiac death (SCD) is a major public health concern worldwide, affecting all age and social groups. Methods: In this retrospective study, of the 8265 autopsies performed in the Institute of Legal Medicine, 1618 cases of SCD were included. The aim of this study is to identify demographic characteristics, etiological factors, epidemiological characteristics and risk factors that lead to SCD. Results: The highest incidence of SCD was in age group 40–69 years (65.0%), 71.6% of this age group being men. Of the total number, 32.1% (520) occurred in the emergency room. The most common cause of sudden death is represented by coronary atherosclerotic disease, reported in 89.8% (1453) of cases, tricoronary lesions being found in 60% (870) of cases. Etiological factors of SCD encountered during autopsies were acute myocardial infarction in 13.9% (225), dilated cardiomyopathy 43.9% (710), cardiac hypertrophy 579 (36.07%), pericarditis 1.9% (30), myocarditis 1.73% (28) and adipositas cordis 5% (81). Along with epicardial fat and BMI, alcohol consumption was recorded in 17.9% (290), this being a potential trigger. Conclusions: Based on forensic autopsy and histological findings, a wide variety of factors are involved in the etiopathogenesis of SCD, some of which can be eliminated through preventive measures implemented early.

## 1. Introduction

In Western countries, approximately 15–20% of the total number of deaths are caused by sudden cardiac death (SCD), this representing a major public health problem. In Romania, there are no data regarding the exact incidence of sudden cardiac death, but in the European Union, approximately a quarter of a million deaths are reported annually and 4 million worldwide [1,2,3,4].

In forensic practice, SCD is the most frequent cause of death from cardiac pathology [2,3,5]. It represents natural death from cardiac causes that occurs through a sudden, unexpected loss of consciousness, occurring immediately or within one hour of the onset of symptoms attributed to a cardiac cause in the presence of witnesses as well as in individuals seen alive 24 h before a death [1,6,7,8,9,10].

In the majority of sudden deaths encountered in forensic practice, there are not enough data regarding a possible symptomatology prior to death, such that the term “unexpected” refers to a death that occurred against the background of apparent health both in a healthy individual and without previously known cardiovascular pathology [11,12,13].

According to Romanian legislation, all deaths occurring at home in individuals without medical documents that can justify a clear cause of death are considered sudden deaths and a forensic autopsy will be mandatory. Also, for all sudden deaths occurring in public places, at workplace or in prisons, a forensic autopsy must be performed.

In terms of etiology, possible causes of sudden cardiac death are acute myocardial infarction, coronary heart disease, hypertrophic and dilated cardiomyopathies, valvular diseases, myocarditis, pericarditis and endocarditis. Identifying the causes and risk factors of SCD is categorically essential, because previous studies have discovered a hereditary arrhythmogenic syndrome in almost half of blood relatives, highlighting that this syndrome can be a possible cause of death, thus identifying living relatives at risk of the same fate.

This study aimed to provide a thorough analysis regarding incidence, circumstances and causes of SCD, highlighting gender and age differences and demographic characteristics in autopsied cases from Mureș County between 2007 and 2017. Another objective is the implementation of specific necessary preventive measures regarding SCD based on the conclusions of this extensive study. 

## 2. Materials and Methods

**Figure 1:**
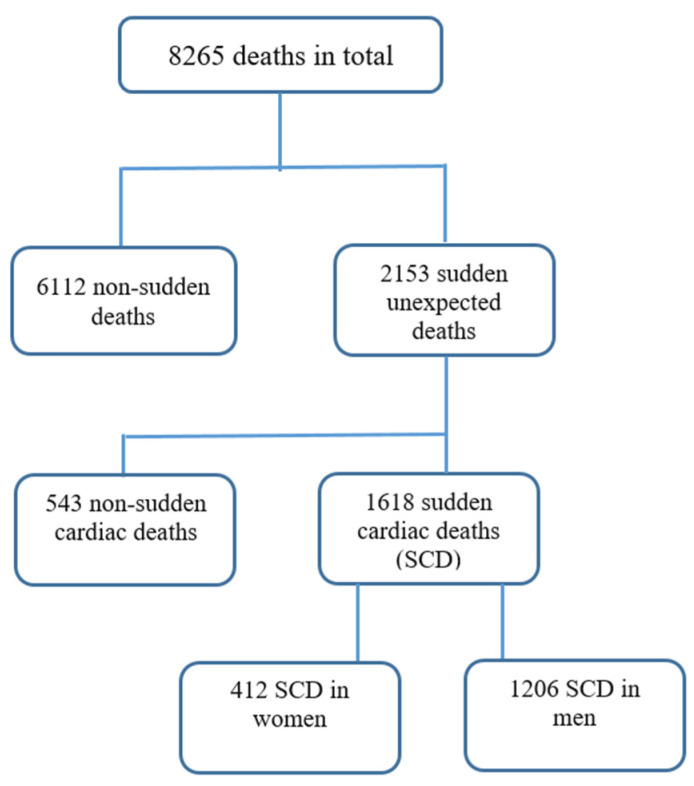
Flowchart of deaths between 2007 and 2017 in ILM of Târgu Mureș.

This is a retrospective single-center study evaluating the complete forensic autopsy reports of the Mureș County Institute of Legal Medicine (ILM), regarding the deaths that occurred in the County between 2007 and 2017. Of the 8265 autopsies performed, 1618 autopsies were included in the study, representing all cases of sudden cardiac death occurring suddenly outside the hospital (home, public place, workplace, penitentiary), as well as cardiac deaths in ambulances and those in the emergency unit in the first hours after arrival (Figure 1). We excluded all violent deaths, all causes of sudden death of non-cardiac origin, deaths with known cardiac pathology, intra- and postoperative death and deaths of newborns and children with cardiac malformations. We also excluded cases if the autopsy report had missing or incomplete data. 

### 2.1. Autopsies

In Târgu Mureș, Romania, the local Institute of Legal Medicine (ILM) represents the responsible authority for performing autopsies on victims of violent and non-violent deaths when necessary to identify and clarify the death cause. A complete forensic autopsy contains demographics data, comorbidities, time and place of the event, resuscitation maneuvers, cause of death, toxicology results and blood alcohol concentration, as well as a histopathological report.

Because the weight of the heart and its macroscopic appearance are important indicators of cardiac disease, all the hearts are autopsied by a standard method: the aorta and pulmonary artery are sectioned 1 cm above their origin; the mediastinal fat and the pericardium are detached, the heart cavities are emptied of postmortem clots, followed by meticulous inspection, measurement and weighing it in fresh state (without prior fixation); this protocol follows all the recommendations of Basso et al. [14].

### 2.2. Statistical Analysis

In this study, we used for statistical analysis of the data both descriptive and inferential statistics. To discriminate between the main pathogenic causes and distribution of lesions at the level of the three main branches of the coronary arteries, we used Fisher exact or Pearson Chi-square tests by using SPSS 20.0 (SPSS, Inc., Chicago, IL, USA). A two tailed *p*-value less than 0.05 was considered statistically significant.

## 3. Results

### Demographic Characteristics and Circumstances of SCD

**Figure 2:**
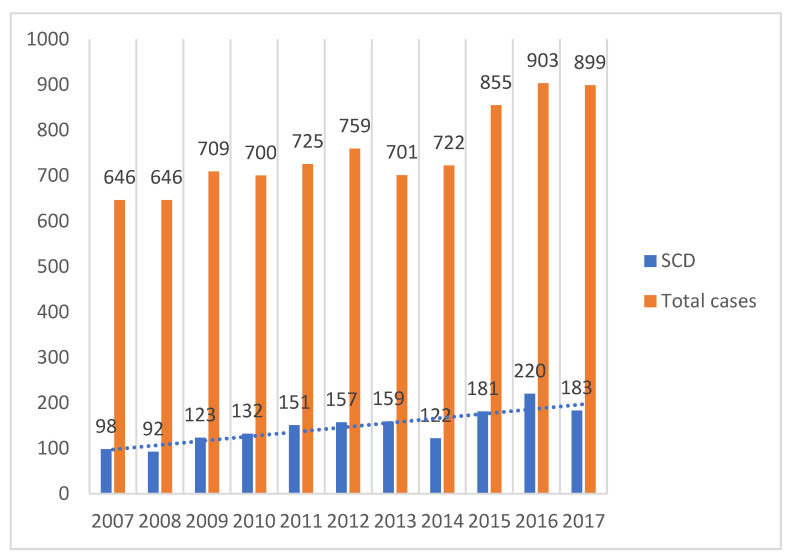
Yearly distribution of SCD.

For the entire duration of the study (2007–2017), a total of 8265 autopsies were performed, of which 1618 autopsies were classified as sudden cardiac deaths (Figure 2) with a tendency to increase in number annually.

Of the total cases of sudden death, an extremely significant percentage is among males 74.5% (1206) compared to females 25.5% (412). The 1618 confirmed SCD cases were divided into three age groups: younger than 39 years (group 1), 40 to 69 years old (group 2) and 70 years old and older (group 3). The highest incidence of SCD in men occurs in the age range 40–69 years, 71.6% (863 cases), while in the female sex a peak-incidence is recorded in the age range 70–99 years, 49.0% (202 cases) (*p* < 0.001). The cases of sudden deaths in young people under 40 years for both genders are also worrying, 6.1% (99) of cases (Table 1).

The environment of origin is relatively similar with a slightly higher distribution in the urban environment 53.52% (866) compared to the rural 46.5% (752). Most sudden deaths occur in the cold season, with the months of December and January recording the most cases; at the opposite pole, there are the months of July and August with 6.1% of cases.

Regarding the place of SCD, 44.6% (722) died suddenly at home, 32.1% (520) died in the emergency services in the first minutes or hours after the onset of symptoms and 21.1% (341) died in a public place (street, bus stations, train station, institutions), 2% (33) at the workplace and 0.1% (2) in the penitentiary. Out of the total number of deaths registered among females, a significant percentage of deaths arose in the emergency department 47.3% (195), compared to the maximum incidence recorded among men 50.3% (848) occurring at home and in public places (*p* < 0.001) (Table 1).

In our study, CAD (coronary atherosclerotic disease) was reported in 1453 cases (89.8%). Performing a gender comparison, coronary atherosclerosis is more common among males 91.5% (1103) compared to females 84.95% (350), *p* = 0.0001. In 38.1% (616) of cases, it is due to atherosclerosis grade IV (stenosis over 75% of the lumen), followed by atherosclerosis grade III (stenosis of 51–75%) in 21.9% (354), atherosclerosis grade II (stenosis between 25–50%) in 15% (243) and grade I atherosclerosis (stenosis of the lumen below 25%) in 14.8% (240). Tricoronary lesions (LAD +LCx + RCA) are the most common, making up 60% (870) of the cases, follow by lesions present in (LAS + LCx) 15.3% (222) and in 13.9% (202) of cases; the lesions are present only in the LAD. The most affected age group is over 70 with a percentage of 67.2% (293) of cases, presenting trivessel lesions (Table 2, sex and age distribution of coronary atherosclerosis). But the atherosclerotic phenomenon does not only affect coronary arteries, it also often extends to the aorta and heart valves. However, valvular disease can have other etiologies, the most frequent being aortic stenosis 2.42% (39) with sex ratio F:M of 2.27:1 (*p* = 0.0009) (Table 2).

Other types of coronary damage found in our cases are as follows: coronary hypoplasia 14.28% (231), coronary bridging 1.98% (32) and coronary thrombosis 6.56% (106) cases.

**Figure 3:**
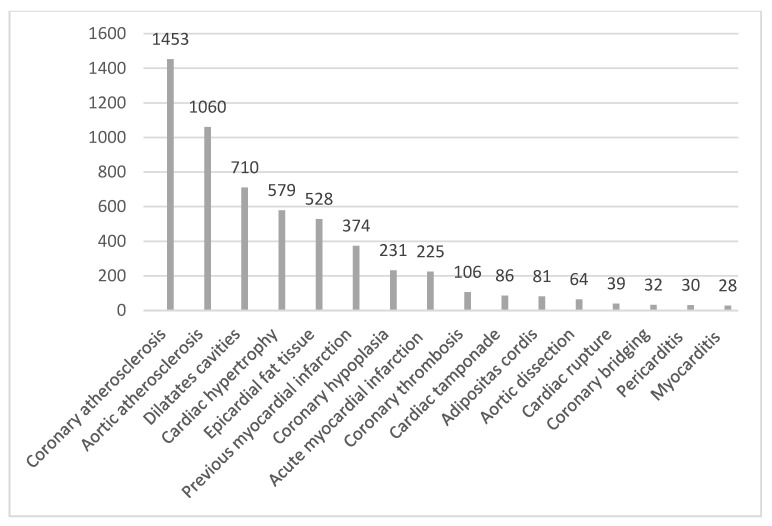
Forensic pathological diagnosis of 1618 SCD cases presented as numbers.

Besides CAD and coronary diseases, many other causes of SCD were detected during autopsies: acute myocardial infarction in 13.9% of cases (225), dilated cardiomyopathy in 43.9% (710), cardiac hypertrophy in 579 cases (36.07%), rupture of aortic root aneurysms in 4% (64), pericarditis in 1.9% (30), myocarditis in 1.73% (28) and adipositas cordis in 5% (81), with the latter affecting 9.5% (39) of all female deaths compared to 3.5% (42) of all male deaths (*p* < 0.0001) (Figure 3).

Among potential SCD triggers, alcohol consumption was recorded in autopsy reports in 17.9% (290) of the total number of cases, being 1.86× more common in men—10.9% (45) of the total number of females compared to 20.3% (245) of the total number of males (*p* < 0.0001). BMI is another risk factor for sudden cardiac deaths, with obesity being predominantly present among women in 38.1% (157) of cases compared to men in whom only 21.2% (256) had a BMI ≥ 30 kg/m^2^, the *p* value being statistically significant (*p* < 0.0001). Compared to the degree of obesity, the most affected was grade I at 14.5% (235) cases, followed by grade II at 8.4% (134) (Table 3).

## 4. Discussion

The incidence of sudden cardiac deaths, according to statistical data carried out at the end of the last millennium, anticipated an increasing trend of heart diseases until the end of 2020 [15,16], an aspect also highlighted in our study, with a considerable increase in the number of cases in recent years (from 15.17% in 2007 reported to the number of yearly medico-legal autopsies performed, to over 20% starting in 2011 and a peak incidence of 24.36% in 2016). However, another study carried out in Romania finds a relatively constant annual rate of SCD in the period 2001–2015 [17].

**Figure 4:**
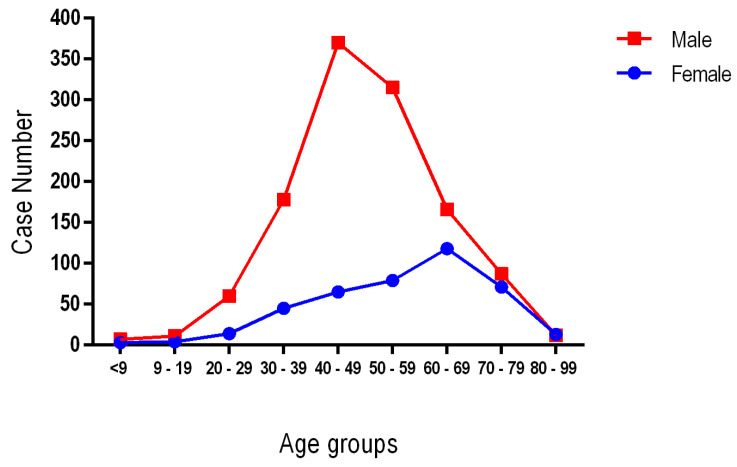
The distribution of age and gender in sudden cardiac death cases.

In this retrospective study conducted over a period of 11 years, among the 1618 cases of SCD registered, 1052 cases (65.0%) were identified in the age group 40–69 years; the youngest age registered was 10 years old and the oldest 97 years old. Although women represent approximately one fourth of all SCD cases, their distribution by age groups shows the highest proportion in the over-70 age group (49.0%), a protective factor being the premenopausal hormonal status as mentioned and Hukilahti et al. [18]. As mentioned, in other worldwide studies such as Braggion-Santos, Sun ZC, Chappex and Winkel, as in our study the incidence of SCD is higher among men, with a sex ratio of 2.92:1; this fact is due to the increased incidence of obstructive coronary artery disease in men (Figure 4) [19,20,21,22].

**Figure 5:**
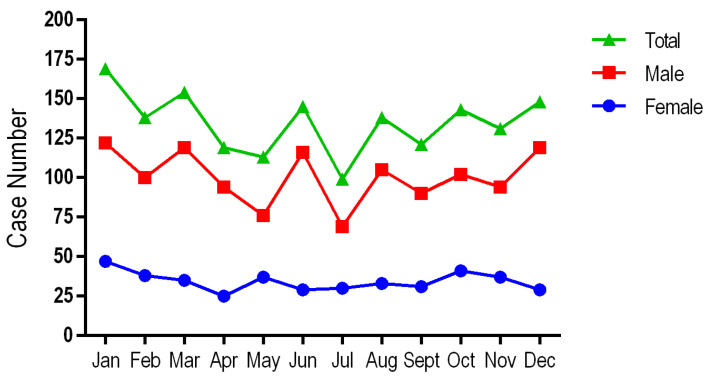
The monthly distribution of sudden cardiac death cases by gender and for the total group.

Looking at the environment of origin, there is a preponderance of SCD in the urban environment (53.52%), this distribution can be correlated with a higher level of development, which brings with it increased daily stress, sedentary lifestyle and pollution [23]. A maximum incidence of sudden cardiac deaths is recorded in the cold season (December, January and February), with a peak incidence in January at 10.6% (172) of cases and a minimum in July at 5.8% (94) of cases, the data being inconsistent with those from Wu Q et al.’s [24] study in which the maximum incidence is recorded in the months of April, May, June and July, but other studies from the Israel and the UK obtained similar data to those from our investigation (Figure 5) [25,26].

As regards the place of death, an overwhelming percentage of 67.8% (1098) occur at home, public places, at work and in the penitentiary, a possible explanation being the brutal onset of cardiovascular symptoms that no longer allows for the provision of emergency care, the data being relatively similar to those of the Braggion-Santos MF study, in which over 50% die at home [19].

It is well known that the most frequent cause of SCD is represented by CAD with a variability of incidence depending on the socio-economic level; thus, in countries such as the UK, the USA, Finland and Sweden similar results to our study are mentioned—89.9% (1453); however, in populations such as Tunisia and Nigeria, the data do not agree with those in developed countries [1,6,21,27,28,29].

Coronary atherosclerosis is presented in 89.8% (1453) of cases, a higher percentage than in the USA, where statistical data show that 80% of SCD was secondary to CAD. With an increased frequency both among the male sex 91.5% (1103) and among the female sex 84.95% (350), (*p* < 0.0001), other studies report a preponderance for men [30,31,32]. Compared to the age groups, in our study atherosclerosis is present most frequently in group 2, 40–69 years, in a proportion of 66.55% (967), followed by age group 3, those over 70 years, 29.94% (*p* < 0.0001). Depending on the degree of coronary atherosclerosis, the vast majority at 38.1% (616) have grade IV atherosclerosis, with stenosis of the lumen between 75 and 100%, with a significant difference between the sexes, only 29.1% (120) of women being affected compared to 41.1% (496) of men presenting the most severe coronary obstruction. Atherosclerosis grade III with stenosis between 50 and 75% is present in 21.9% (354) and grade II with 25–50% stenosis in 14.6% (60) with a similar gender distribution. Grade I atherosclerosis affects 14.8% (240) of cases, with a higher frequency in the female sex, 19.2% (79) of all women. In contrast to other studies such as the ones from Denmark [33] and China [24], in which obstruction of a single coronary artery is most frequently present (LAD being the most frequently obstructed artery), in the group studied by us, tricoronary lesions overwhelmingly receive 60% (870), and the worrying fact is that even in the first age group under 40 years, 39.2% (20) require additional preventive measures for young people. In the previously mentioned studies as well as in many others, the obstruction of a single coronary branch is present with a higher incidence in women, and tricoronary lesions in men; in our study, no significant differences between the sexes are recorded in terms of coronary obstruction. The LAD is the most affected vessel, both in our and other studies [34,35]. Even though it is known that coronary atherosclerosis presents an increased incidence with age, the sedentary lifestyle in the urban environment correlated with the unhealthy eating habits to which the youth are exposed today have led to an increase in the incidence of coronary atherosclerosis which in the case of our study is more than half, 51.5% (51) of those under 40 years of age.

Coronary thrombosis is a complication of coronary atherosclerosis through the erosion and rupture of the atheromatous plaque, which causes SCD in 6.56% (106) of the entire analyzed group, with the most significant percentage of 11.1% (11) in young people under 40 years old, the most frequently affected vessel being also the LAD. In 65.5% (1050) of cases, CAD is associated with varying degrees of aortic atherosclerosis: 41.2% (437) grade I, 25.4% (269) grade II and 33.4% (354) grade III. Another segment affected by atherosclerosis is the heart valves, with a percentage of 8.4% (134) of cases of valvular atherosclerosis being recorded (Table 4).

Myocardium bridging is a congenital anomaly in which a segment of one or more coronary arteries passes through the myocardial muscle like a tunnel instead of sitting on its surface. Thus, coronary bridging can cause vascular compression, causing associated hemodynamic changes such as angina, acute coronary syndrome and arrhythmias, but most of them are asymptomatic, being a cause of sudden cardiac death, discovered after autopsy [36]. Cardiac hypertrophy is the most common disease of the heart muscle, affecting 0.2–0.5% of the general population; the disease can evolve into heart failure [37]. It is present in 1.98% (32 cases), mostly among males, with age group 2 presenting the highest incidence at 22 cases, and with the predominant damage being to the LAD, an aspect also highlighted in the study by Loukas [38]. According to the data from the literature, the incidence of myocardium bridging varies depending on the angiographic diagnostic method or its detection during the autopsy. We found a similar incidence to another study from India, but the diagnostic method they used was angiographic, and the incidence was much lower compared to other studies [39].

Even though according to literature data, congenital anomalies of the coronary arteries are rare, affecting 0.3–1.3% of the general population, they are a cause of myocardial ischemia and sudden death especially in young people [40]. Of the three age groups in our study, deaths caused by coronary hypoplasia present an extremely high incidence at 21.2% (21) among those aged ≤ 39 years, but also in the other two age groups, with 14.7% (155) in those in age group 2 and 14.3% (55) in those over 70 years old, the explanation being the development with age of the collateral arterial branches that supplement coronary circulation (Table 4).

Coronary thrombosis does not play a major role in sudden cardiac deaths, an aspect also highlighted in our study, in which only 6.56% (106) of cases presented coronary thrombosis, but it is worth analyzing the fact that it had an almost double incidence in those aged under 40 years, at 11.1% (11), data inconsistent with another study carried out in the past, in which the incidence of coronary thrombosis was increased in those aged between 40 and 59 years [41]. The increase in the number of cases of coronary thrombosis in young people in the last 3 decades is explained by the emergence of risk factors and implicitly the development of early coronary atherosclerosis.

The second cause of SCD is due to cardiomyopathies, most occurring consecutively to dilated cardiomyopathy at 43.9% (710) of cases, without significant differences between sexes; with an average across genders of 36.07% (579), hypertrophic cardiopathy predominantly affects male in 38.58% (461) of cases and females in 28.78% (118) of cases (*p* < 0.001). Another study from France found different data, showing no cases of hypertrophic cardiopathy in women, and a double incidence of dilated cardiopathy in women. Heart weight was over 500 g in 42.2% (174) of women and 57.1% (689) of men (*p* < 0.001) with weight being greater in those with hypertrophic cardiomyopathy, the data being similar to other studies [42].

Another retrospective study of sudden cardiac deaths conducted from 2005 to 2014 in China that included a cohort of 769 SCDs showed an extremely low percentage of cardiomyopathies of only 23 cases, but with an increased percentage of myocarditis [43] compared to our study in which the latter had an incidence of 1.73% (28) cases, with pericarditis affecting 1.9% (30) of cases, both with an increased frequency in the 40–69 year old age group (Table 5).

Myocardial fibrosis is considered a trigger that can cause sudden cardiac death in about 90% of cases according to the study by Prati et al. [44]; in our case, it has an incidence of 77.3% (1250), *p* < 0.0001, with a similar distribution between sexes but with a predominance in age group 3 at 83.3% (389), closely followed by age group 2 at 77.2% (812) and with almost half being affected among those under 40 years old.

Previous myocardial infarction previously undetected clinically was present in 23.11% (374) of cases, with the most frequent location at the level of the posterior wall at 10% (161), followed by the lateral wall at 6.4% (104), the anterior wall at 4.3% (69) and at the septal level in 2.5% (40) of cases.

Acute myocardial infarction is a coronary syndrome characterized by an ischemia of sufficient duration to cause irreversible damage to the affected myocytes. In the production of acute myocardial infarction, the determining factor is represented by the reduction of coronary flow secondary to acute coronary obstruction; as such, it is possible that the first manifestation is sudden death. Of the autopsies included in our study, only 13.9% (225) were due to acute myocardial infarction; this represents a low percentage compared to other studies in which it exceeded 50% [45]. Compared to the old myocardial infarction, the most frequent location was at the level of the anterior wall at 32% (72), the lateral wall at 30.7% (69) and the posterior wall at 27.6% (62).

Acute myocardial infarction survivors are at risk of sudden death [46]. Analyzing the predisposition of acute myocardial infarction in relation to sex, 15.5% (65) are women and 13.3% (161) are men; in the case of previous myocardial infarction, 19.17% (79) are women and 24.46% (295) are men; so it can be seen that in the case of women, acute myocardial infarction represents the immediate cause of sudden death.

Another cause of sudden death is cardiac tamponade at 5.3% (86), which occurs most often, following acute myocardial infarction through non-traumatic rupture of the heart [47]. It is present in 2.41% (39) of cases, with the ruptured wall being the right ventricular wall, with a slightly higher incidence in women of 2.92% (12) compared to 2.24% (27) in men in the over 70 year old age group. Similar results were found in other studies [48], but in other studies the incidence is higher in males [49]. Non-traumatic rupture of the aorta represents another cause of sudden death with preferential damage to the thoracic aorta in the ascending portion, with an increased frequency of 4% (64), the percentage between sexes being statistically insignificant. 

A diagnostic challenge is represented by adipositas cordis, the symptom of which is most often SCD, not being accompanied by previous clinical manifestations; the existing paraclinical examinations are non-specific, a fact that leads to its identification post-mortem, during the autopsy [50]. In 9.5% (39) of women, it was detected macroscopically and on hematoxylin-eosin sections, compared to 3.5% (42) of men, *p* < 0.001, with age group 1, ≤39 years, being the most affected at 10.1% (10) of cases followed by group 3 with 5.4% (25) and group 2 with 4.4% (46), *p* = 0.004. A possible explanation for the predominance of women could be the redistribution of visceral fat within Cushing’s syndrome, which is known to have an increased prevalence in women [51].

Myocarditis is an inflammation of the myocardium that can cause sudden death. In a study conducted in the State of Maryland between 2005 and 2014, its incidence was 0.70%, with an increased frequency in those under 30 years of age [52]. Although, according to other studies, myocarditis represents 20% of sudden deaths among young people, in our study of all sudden deaths, myocarditis confirmed by histopathological examination was recorded in 1.73% (28) of cases with relatively similar involvement of the three age groups. In the case of pericarditis, the data are similar [53].

However, apart from the direct mechanisms that can lead to SCD, there are also favoring factors such as epicardial fat tissue, BMI, and positive values of alcoholaemia that have additional negative effects in thanatogenesis. In 32.65% (528) of the total cases, an increase in epicardial fat tissue was detected with relatively equal damage to all three age groups [54].

BMI is another risk factor for sudden cardiac deaths, obesity being predominantly present among women in 38.1% (157) of cases compared to men, in whom only 21.2% (2565) have a BMI ≥ 30 kg/m^2^; the values are statistically significant (*p* < 0.0001) compared to the degree of obesity, the most frequent being degree I (BMI 30–34.9 kg/m^2^) 14.5% (235) cases, followed by degree II (BMI 35–39.9 kg/m^2^) 8.4% [55].

Of a total of 290 cases of positive blood alcohol levels, a percentage of 84.48% (245) is found in the male sex compared to 15.52% (45) of cases of positive blood alcohol levels detected in the female sex (*p* < 0.001); similar data are also mentioned in the literature [56,57].

## 5. Conclusions

Sudden cardiac death still remains a major public health concern with socio-economic impact, affecting all age groups, not just the elderly population, and both sexes but to different degrees. Based on forensic autopsy findings, a wide variety of factors are involved in the etiopathogenesis of SCD, some of which can be eliminated through preventive measures implemented early. A very important aspect in the prevention of SCD is the identification of the population at risk and its protection by implementing effective monitoring and treatment protocols with a high diagnostic effort from specialists. Because SCD affects males three times more often than females (comparable results are observed in other studies), it is necessary to carry out mandatory periodic monitoring through primary medicine network (general practitioner) with a predominance of men, even young people. Periodic additional clinical and paraclinical investigations are also welcome in order to reduce the risk of death from SCD.

## Figures and Tables

**Table 1:** Demographic characteristic and circumstance of SCD.

Parameter		All Cases (*n* = 1618)	Female Sex (*n* = 412, 25.5%)	Male Sex (*n* = 1206, 74.5%)	*p* Value	Group 1 ≤39 Years (*n* = 99)	Group 2 40–69 Years (*n* = 1052)	Group 3 ≥70 Years (*n* = 467)	*p* Value
Age groups	10–39 years	99 (6.1%)	21 (5.1%)	78 (6.5%)	<0.0001	99 (6.1%)	1052 (65.0%)	467 (28.9%)	<0.0001
	40–69 years	1052 (65.0%)	189 (45.9%)	863 (71.6%)					
	70–99 years	467 (28.9%)	202 (49.0%)	265 (22%)					
Urban environment		866 (53.52%)	207 (50.24%)	659 (54.64%)	0.12	46 (46.5%)	559 (53.1%)	261 (55.9%)	0.21
Death in winter		456 (28.2%)	115 (27.9%)	341 (28.3%)	0.75	28 (28.3%)	287 (27.3%)	141 (30.2%)	0.51
Place of death	Home	722 (44.6%)	170 (41.3%)	552 (45.8%)	<0.0001	42 (42.4%)	524 (49.8%)	156 (33.4%)	<0.0001
	Emergency Reception Unit	520 (32.1%)	195 (47.3%)	325 (26.9%)		28 (28.3%)	243 (23.1%)	249 (53.3%)	
	Public place	341 (21.1%)	45 (10.9%)	296 (24.5%)		28 (28.3%)	252 (24%)	61 (13.1%)	
	At work	33 (2.0%)	2 (0.5%)	31 (2.6%)		1 (1.0%)	31 (2.9%)	1 (0.2%)	
	Penitentiary	2 (0.1%)	0 (0%)	2(0.2%)		0 (0.0%)	2 (0.2%)	0 (0%)	

**Table 2:** Distribution of coronary atherosclerotic lesions.

Parameter		All Cases (*n* = 1618)	Female Sex (*n* = 412, 25.5%)	Male Sex (*n* = 1206, 74.5%)	*p* Value	Group 1 ≤39 Years (*n* = 99)	Group 2 40–69 Years (*n* = 1052)	Group 3 ≥70 Years (*n* = 467)	*p* Value
Coronary atherosclerosis		1453 (89.8%)	350 (84.95%)	1103 (91.5%)	0.0001	51 (51.5%)	967 (91.9%)	435 (93.1%)	<0.0001
Degree of coronary atherosclerosis	Grade I	240 (14.8%)	79 (19.2%)	161 (13.3%)	0.001	15 (29.4%)	166 (17.2%)	59 (13.6%)	
	Grade II	243 (15%)	60 (14.6%)	183 (15.2%)		11 (21.6%)	157 (16.2%)	75 (16.1%)	
	Grade III	354 (21.9%)	91 (22.1%)	263 (21.8%)		9 (17.6%)	241 (24.9%)	104 (23.9%)	
	Grade IV	616 (38.1%)	120 (29.1%)	496 (41.1%)		16 (31.3%)	403 (41.7%)	197 (42.2%)	
Coronary atherosclerotic lesion location	LAD	202 (13.9%)	46 (13.1%)	156 (14.2%)	0.37	14 (27.5%)	142 (14.7%)	46 (10.6%)	0.001
	LCx	66 (4.6%)	10 (2.9%)	56 (5.1%)		5 (9.8%)	49 (5.1%)	12 (2.8%)	
	RCA	90 (6.2%)	20 (5.7%)	70 (6.4%)		4 (7.8%)	63 (6.5%)	23 (5.3%)	
	LAD + LCX + RCA	870 (60%)	222 (63.4%)	648 (58.9%)		20 (39.2%)	557 (57.8%)	293 (67.2%)	
	LAD + LCX	222 (15.3%)	52 (14.9%)	170 (15.5%)		8 (15.7%)	156 (15.8%)	61 (14.0%)	

**Table 3:** Triggers of SCD.

Parameter		All Cases (*n* = 1618)	Female Sex (*n* = 412, 25.5%)	Male Sex (*n* = 1206, 74.5%)	*p* Value	Group 1 ≤39 Years (*n* = 99)	Group 2 40–69 Years (*n* = 1052)	Group 3 ≥70 Years (*n* = 467)	*p* Value
Body Mass Index, kg/m^2^	Underweight (≤18.49)	151 (9.3%)	37 (9.0%)	114 (9.5%)	<0.0001	6 (6.1%)	84 (8.0%)	61 (13.1%)	0.01
	Normal weight (18.5–24.9)	1054 (65.1%)	218 (52.9%)	836 (69.3%)		72 (72.7%)	695 (66.1%)	287 (61.5%)	
	Obesity (≥30)	413 (25.5%)	157 (38.1%)	256 (21.2%)		21 (21.2%)	273 (38.1%)	119 (25.5%)	
Degree of obesity	Grade I (BMI 30–34.9)	235 (14.5%)	71 (17.2%)	164 (13.6%)	<0.0001	14 (14.1%)	149 (14.2%)	72 (15.4%)	0.23
	Grade II (BMI 35–39.9)	134 (8.3%)	64 (15.5%)	70 (5.8%)		3 (3.0%)	91 (8.7%)	40 (8.6%)	
	Grade III (BMI > 40)	44 (2.7%)	22 (5.3%)	22 (1.8%)		4 (4.0%)	33 (3.1%)	7 (1.5%)	
The presence of alcohol in the blood		290 (17.9%)	45 (10.9%)	245 (20.3%)	<0.0001	17 (17.2%)	222 (21.1%)	51 (10.9%)	0.001

**Table 4:** Comparison of autopsy findings between female and male sex in the entire studied population.

Parameter		All Cases (*n* = 1618)	Female Sex (*n* = 412, 25.5%)	Male Sex (*n* = 1206, 74.5%)	*p* Value
Coronary bridging, *n*		32 (1.98%)	5 (1.21%)	27 (2.24%)	0.69
Coronary hypoplasia, *n*		231 (14.28%)	69 (16.75%)	162 (13.43%)	0.13
Coronary thrombosis, *n*		106 (6.56%)	22 (5.34%)	84 (6.97%)	0.75
Aortic atherosclerosis, *n*		1060 (65.5%)	279 (67.5%)	781 (64.9%)	0.71
Aortich atherosclerosis degree, *n*	Incipient	437 (41.2%)	118 (28.6%)	319 (26.5%)	0.71
	Moderate	269 (25.4%)	73 (17.7%)	196 (16.3%)	
	Severe	354 (33.4%)	88 (21.4%)	266 (22.1%)	
Aorta dissection, *n*		64 (4%)	18 (4.4%)	46 (3.80%)	0.61
Valvular atherosclerosis, *n*		134 (8.4%)	48 (11.9%)	86 (7.2%)	0.004
Aortic stenosis, *n*		39 (2.42%)	17 (4.1%)	22 (1.8%)	0.009
Dilates cavities, *n*		710 (43.9%)	172 (41.75%)	538 (44.6%)	0.31
Cardiac hypertrophy, *n*		579 (36.07%)	118 (28.78%)	461 (38.58%)	0.001
Epicardial fat tissue, *n*		528 (32.65%)	127 (30.8%)	401 (33.25%)	0.15
Adipositas cordis, *n*		81 (5%)	39 (9.5%)	42 (3.5%)	<0.0001
Pericarditis, *n*		30 (1.9%)	8 (1.9%)	22 (1.8%)	0.87
Myocardiosclerosis, *n*		1250 (77.3%)	313 (76%)	937 (77.7%)	0.47
Myocarditis, *n*		28 (1.73%)	6 (1.46%)	22 (1.82%)	0.62
Cardiac tamponade, *n*		86 (5.3%)	22 (5.3%)	64 (5.3%)	0.97
Cardiac rupture, *n*		39 (2.41%)	12 (2.92%)	27 (2.24%)	0.18
Acute myocardial infarction, *n*		225 (13.9%)	64 (15.5%)	161 (13.3%)	0.26
Location of AMI	Cardiac septum, *n*	22 (9.8%)	4(6.2%)	18 (11.2%)	0.35
	Posterior wall, *n*	62 (27.6%)	18 (28.1%)	44 (27.3%)	
	Lateral wall, *n*	69 (30.7%)	20 (31.2%)	49 (30.4%)	
	Anterior wall, *n*	72 (32%)	22 (34.4%)	50 (31.1%)	
Previous myocardial infarction, *n*		374 (23.11%)	79 (19.17%)	295 (24.46%)	0.02
Location of previous MI	Cardiac septum, *n*	40 (2.5%)	6 (1.5%)	34 (2.8%)	0.05
	Posterior wall, *n*	161 (10%)	33 (8%)	128 (10.6%)	
	Lateral wall, *n*	104 (6.4%)	29 (7%)	75 (6.2%)	
	Anterior wall, *n*	69 (4.3%)	11 (2.7%)	58 (4.8%)	
Heart weight, median (IQR)		500 (420–591.25)	457.5 (375–540)	500 (440–600)	<0.0001
Heart weight	<500 g, *n*	755 (46.7%)	238 (57.8%)	517 (42.9%)	<0.0001
	≥500 g, *n*	863 (53.3%)	174 (42.2%)	689 (57.1%)	

**Table 5:** Comparison of autopsy findings between age groups in the entire studied population.

Parameter		Group 1- ≤39 Years (*n* = 99)	Group 2- 40–69 Years (*n* = 1052)	Group 3- ≥70 Years (*n* = 467)	*p* Value
Coronary bridging, *n*		3 (2.0%)	22 (2.1%)	8 (1.7%)	0.03
Coronary hypoplasia, *n*		21 (21.2%)	155 (14.7%)	55 (14.3%)	0.26
Coronary thrombosis, *n*		11 (11.1%)	64 (6.1%)	31 (6.6%)	0.12
Aortic atherosclerosis	Incipient	14 (14.1%)	318 (30.2%)	105 (22.5%)	<0.0001
	Moderate	3 (3.0%)	184 (17.5%)	82 (17.6%)	
	Severe	1 (1.0%)	173 (16.4%)	180 (38.5%)	
Aorta dissection, *n*		7 (7.1%)	37 (3.5%)	20 (4.3%)	0.20
Valvular atherosclerosis, *n*		134 (8.4%)	48 (11.9%)	86 (7.2%)	0.004
Aortic stenosis, *n*		39 (2.42%)	17 (4.1%)	22 (1.8%)	0.009
Dilates cavities, *n*		36 (36.4%)	460 (43.7%)	214 (45.8%)	0.22
Cardiac hypertrophy, *n*		35 (35.4%)	382 (36.3%)	164 (35.1%)	0.89
Epicardial fat tissue, *n*		32 (32.3%)	360 (34.2%)	137 (29.3%)	0.41
Heart lipomatosis, *n*		10 (10.1%)	46 (4.4%)	25 (5.4%)	0.04
Pericarditis, *n*		3 (3.0%)	20 (1.9%)	7 (1.5%)	0.58
Myocarditis, *n*		3 (3.0%)	21 (2.0%)	4 (0.9%)	0.17
Myocardiosclerosis, *n*		49 (49.5%)	812 (77.2%)	389 (83.3%)	<0.0001
Cardiac rupture, *n*		1 (1.0%)	24 (2.4%)	16 (3.4%)	0.47
Cardiac tamponade, *n*		6 (6.1%)	57 (5.4%)	3 (4.9%)	0.87
Acute myocardial infarction, *n*		13 (13.1%)	135 (12.8%)	77 (16.5%)	0.16
Previous myocardial infarction, *n*		12 (12.1%)	255 (24.2%)	107 (22.9%)	0.02
Acute pulmonary edema, *n*		68 (68.7%)	631 (60.0%)	305 (65.3%)	0.05

## Data Availability

The original contributions presented in the study are included in the article, further inquiries can be directed to the corresponding authors.
